# Primary and “Pre-Primary” Aldosteronism in Resistant Hypertension: A Practical, Pragmatic, and Prudent Approach in Resource-Limited Milieu

**DOI:** 10.7759/cureus.72161

**Published:** 2024-10-22

**Authors:** Yug Garg, Madhumati S Vaishnav, Nidhi Garg, Reshma B Vijay, Leena Lekkala, Siddhartha Dinesha, Pushpa Ravikumar, Thummala Kamala, Kavitha Muniraj, Sathyanarayana Srikanta

**Affiliations:** 1 Endocrinology, Diabetes and Metabolism, Samatvam Endocrinology Diabetes Center, Samatvam: Science and Research for Human Welfare Trust, Bangalore, IND

**Keywords:** aldosterone-renin ratio, estimated glomerular filtration rate, mineralocorticoid receptor antagonists, plasma renin activity, pre-primary aldosteronism, primary aldosteronism, resistant hypertension, resource limitations, serum aldosterone, serum creatinine

## Abstract

Introduction

Primary aldosteronism (PA), once considered rare, is now recognized as the most common cause of secondary hypertension, accounting for almost a quarter of resistant hypertension (RH) cases. Despite this, PA remains underdiagnosed, with an extremely low percentage of RH patients undergoing screening.

Methods

In a specialty diabetes-endocrinology clinic, the aldosterone:renin ratio (ARR) was assessed in 115 consecutive RH patients (ages 21-93 years; 47% male; 87% with type 2 diabetes). Fasting blood samples were drawn in a standing position after 30 minutes of walking. Adrenal imaging (CT/MRI) was performed for those with an ARR >20.

Results

ARR values ranged from 0.4 to 227 (ARR <10 (35%); 11-20 (19%), 21-40 (25%), and >40 (21%)), with corresponding stepwise decreasing plasma renin activity (PRA) (P= 1E-6) and increasing serum aldosterone (SA) (P= 8E-7). Increasing ARR tended to be associated with an increase in serum creatinine (R= 0.23; P= 0.03) and a decrease in estimated glomerular filtration rate (eGFR) (R= -0.24; P= 0.02) and an increase in urine albumin: creatinine ratio. The ARR> 40 group displayed the highest serum creatinine, lowest eGFR, higher urine albumin: creatinine ratio, highest serum sodium, lowest serum potassium, and highest (44%) abnormal adrenal imaging (bilateral hyperplasia diffuse/nodular; solitary adenoma), reflecting a later stage of the pathological spectrum. PA treatment with mineralocorticoid receptor antagonists (MRAs) had a salutary effect.

Conclusions

Our observations further reinforce that PA is not a binary condition, but exists as a spectrum disorder responsive to MRAs, even in patients with mildly elevated or normal aldosterone levels. Early disease detection/recognition (“renin-independent aldosterone production”) can be facilitated by marking “pre-primary" aldosteronism (ARR 11-20), followed by monitoring progression (periodic rescreening) and optimizing treatment, with hopeful mitigation of end-organ damage in RH.

## Introduction

Primary aldosteronism (PA) was historically considered a rare cause of hypertension, but contemporary research has redefined it as the most common etiology of secondary hypertension. Epidemiological studies estimate its prevalence at approximately 20% among patients with resistant hypertension (RH), 10% in those with severe hypertension, and 6% in individuals with otherwise uncomplicated hypertension. Despite these significant figures, PA remains vastly underdiagnosed and undertreated, with a screening rate reported at a mere 2.1% in RH patients [1-6].

This disparity between prevalence and diagnosis underscores a critical gap in clinical practice and highlights the need for enhanced screening protocols. The first step in the diagnosis of PA is case detection and involves testing patients who are at risk, including individuals with RH, as well as those with, well-controlled hypertension, a first-degree relative with PA, hypokalemia, an adrenal nodule, atrial fibrillation, obstructive sleep apnea, or a family history of an early stroke (i.e., younger than 40 years). The current diagnostic approach primarily relies on the aldosterone:renin ratio (ARR), with a positive screening threshold typically set at 20-30 ng/dL:ng/mL/hour using plasma renin activity (PRA).

Compared to primary hypertension, PA is associated with greater end-organ damage and a higher risk of cardiovascular morbidity, including heart failure, stroke, myocardial infarction, and atrial fibrillation. This underscores the importance of early detection and targeted treatment. Effective treatment with mineralocorticoid receptor antagonists (MRAs) not only improves blood pressure control but also reduces cardiovascular risk. As mentioned, despite the clear benefits of early detection and targeted therapy, the screening rates for PA remain low, and MRAs are underutilized in the management of hypertension. This gap in clinical practice highlights the need for increased awareness and routine screening for PA among hypertensive patients. All physicians managing hypertension should be vigilant in screening appropriate patients for PA to ensure timely and effective intervention.

Pathophysiology and evolution of PA

Recent insights into the pathophysiologic continuum of PA suggest a disease spectrum that challenges the binary classification of PA and normotension. This spectrum includes a variety of adrenal pathologies such as bilateral adrenal adenomas, unilateral hyperplasia, micronodules, and microscopic aldosterone-producing cell clusters (APCCs). Notably, PA can manifest in patients with RH and may present with mildly elevated or even ostensibly normal aldosterone levels. These patients can still benefit from treatment with mineralocorticoid receptor (MR) antagonists, reflecting the broad and sometimes subtle clinical presentations of PA [7].

Clinical implications

Thus, the continuum nature of PA pathology implies that traditional binary diagnostic approaches may be inadequate. Current ARR thresholds used for screening may fail to identify a significant number of patients who fall within the lower range of this spectrum. These patients, despite having lower ARRs, could still be experiencing adverse effects from hyperaldosteronism (relative mineralocorticoid excess) and might benefit from therapeutic interventions. Therefore, defining additional lower ARR thresholds to mark early stages of PA pathogenesis (similar to prediabetes, impaired fasting glucose and impaired glucose tolerance, and prehypertension), could facilitate early disease identification and more precision treatment (with MR antagonists), even before the development of full-blown disease: overt PA (similar to type 2 diabetes).

The Indian scenario (a resource-limited setting)

Resource limitations and reduced awareness among the medical community are some of the additional challenges in economically underprivileged countries. In India, the prevalence, clinical characteristics, and management of PA have been under-explored compared to Western populations. A 2024 editorial by Memon and Bandgar summarised the Indian scenario and emphasised the need for increasing awareness about PA screening tests and their interpretation [8].

A prospective outpatient diabetes clinic study from western India involving cohorts with type 2 diabetes and hypertension reported a PA prevalence of at least 4.1%, with estimates reaching as high as 8.7% [9]. Patients with positive screening tests had a higher proportion of females, frequent history of hypertensive crises, uncontrolled blood pressure, diagnosis of hypertension before diabetes, and higher systolic and diastolic blood pressures. Patients with positive confirmatory tests had longer duration of diabetes and hypertension and higher creatinine.

In a study conducted in North India, PA was diagnosed in 17.8% of young individuals (age of hypertension onset <40 years) with hypertension and referred to endocrinology and cardiology clinics for evaluation of secondary hypertension [10]. The prevalence of PA increased with the grade of hypertension, number of antihypertensive medications, and severity of hypokalaemia.

With this background, the current study aimed to retrospectively analyse our real-life experiences of using ARR for routine screening for PA, in consecutive subjects presenting with RH of undetermined etiology at a specialty endocrinology diabetes clinic in India.

## Materials and methods

This was a retrospective chart review study from 2018 to 2023 conducted at a specialty endocrinology diabetes clinic in India, focusing on patients with RH. This clinic serves both as a primary care center and a tertiary referral center, serving the entire socioeconomic spectrum of the community, in Bangalore, Karnataka, India. A total of 115 consecutive patients diagnosed with RH of undetermined etiology were reviewed.

The study was conducted in accordance with the Declaration of Helsinki. It was approved by the institutional review board of the specialty clinic. The research protocol was approved by the local ethics committee, Science for Health (reference number: 2022-10-06-01; dated October 22, 2022). Written informed consent was deemed as not necessary as the study involved retrospective data analysis - medical record review only.

Inclusion and exclusion criteria

Patients included in the study met the following criteria: (a) Diagnosis of RH; (b) never treated with MRAs; (c) Age above 20 years; and (d) Undergone screening for PA and subsequent investigations. Patients were excluded if they had a known diagnosis of PA before the study.

Definition of RH

In accordance with guidelines, RH was defined as the blood pressure (BP) of a hypertensive patient that remained elevated above the target level despite the concurrent use of three antihypertensive agents of different classes. These typically included a long-acting calcium channel blocker (CCB), a renin-angiotensin system blocker (angiotensin-converting enzyme (ACE) inhibitor or angiotensin receptor blocker (ARB)), and a diuretic. All agents were administered at their maximum tolerated doses and at the appropriate dosing frequency. RH also included patients whose BP achieved target values after the addition of a fourth or more antihypertensive medication (controlled RH).

Screening procedure

To identify potential cases of PA, ARR was measured in all participants. The screening procedure was very strictly standardized to ensure accuracy and consistency. Blood samples were drawn from patients while they were standing, after 30 minutes of walking, to stimulate maximum renin release. Samples were collected in the overnight fasting state. However, for practical considerations, no alterations were done with respect to prior antihypertensive medications, such as beta-blockers, ACE inhibitors, ARBs, and diuretics, which can affect renin and aldosterone levels, leading to false-positive or false-negative results.

Laboratory analysis

Serum aldosterone and PRA were measured using chemiluminescent immunoassay (CLIA) methods. Reference ranges for serum aldosterone were 2.52-39.2 ng/dL when upright and 1.76-23.2 ng/dL when supine. Reference ranges for PRA were 0.15-2.33 ng/mL/hour in the lying position and 0.10-6.56 ng/mL/hour in the standing position. The ARR was calculated using the formula: ARR = serum aldosterone (ng/dL)/PRA (ng/mL/hour). An ARR value greater than 20 was considered a positive screen for further investigation.

Diagnostic follow-up

Patients with an ARR >20 underwent additional diagnostic imaging to assess adrenal morphology. Computed tomography (CT) was performed to identify adrenal adenomas, hyperplasia, or other abnormalities.

Data collection

Clinical data, including patient demographics, medical history, and clinical presentation were recorded. Specific attention was given to cases presenting with hypokalemia, adrenal incidentalomas, or other symptoms suggestive of PA.

Statistical analysis

Descriptive statistics (ANOVA, chi-squared test, correlations) were used to summarize the prevalence and characteristics of PA in the study population. Regression analysis was conducted for the evaluation of biomarker interrelationships using Microsoft Excel (2019; Microsoft Corporation, Redmond, Washington, United States).

## Results

Demographic characteristics

A total of 115 consecutive patients with RH were included in the study (after ruling out other potential causes for RH like renal parenchymal or renovascular disease). The age of the patients at the time of evaluation ranged from 21 to 93 years, with a mean age of 60 ± 16 years. Male patients comprised 47% of the participants in the cohort. The mean duration of hypertension was 12.9 ± 10.6 years. The mean highest-ever systolic blood pressure was 174 ± 30 and diastolic blood pressure was 102 ± 20 mm Hg. Mean number of anti-hypertensive medications at baseline was 3.8 ± 0.6 in this cohort (Table 1).

**Table 1:** Salient clinical and biochemical features of the patients with resistant hypertension evaluated in the study, grouped according to increasing ARR (N= 115). The ARR values among the patients varied widely, ranging from 0.4 to 227. The distribution of ARR values is detailed herewith, categorizing the patients into distinct groups to facilitate analysis (Group A: ARR < 10: 35% of patients; Group B: ARR 11-20: 19%; Group C: ARR 21-40: 25%; and Group D: ARR > 40: 21%). Overall, 46% of the patients had an ARR >20, indicating a positive screen for primary aldosteronism (PA). Reference ranges: PRA (ng/ml/hour): lying position: 0.15-2.33, standing position: 0.10-6.56; S Aldosterone (ng/dL): upright: 2.52-39.2, supine: 1.76-23.2; S Creatinine (mg/dl): women 0.55-1.02, men 0.72-1.18 Note: OSA (%): As part of routine clinical care, a home sleep study was performed only in the subgroup of RH subjects (N= 20 + 10 + 15 + 15= 60) who exhibited clinical symptoms suggestive of possible OSA. Data presented as means±SDs, and frequency (percentage) where indicated; P values for comparison of the four ARR groups were calculated using one-way ANOVA (for means ± SDs) and chi-square test (for counts or numbers). In all analyses, P<0.05 was considered statistically significant; not significant (P≥0.05); Next to the p-values reported in the table, the test statistics (F-value for ANOVA and χ²-value for the chi-square test) have been added together with their corresponding degrees of freedom. ARR: aldosterone renin ratio; PRA: plasma renin activity; S: serum; eGFR: estimated glomerular filtration rate; HTN: hypertension; SBP: systolic blood pressure; DBP: diastolic blood pressure; A:C: albumin:creatinine; ECHO EF%: echocardiography ejection fraction%; OSA: obstructive sleep apnea; AHI: apnoea hypopnea index; IHD: ischemic heart disease; RH: resistant hypertension

Biomarker	Reference Range	Units	ARR 0-10	ARR 11-20	ARR 21-40	ARR >40	Test Statistic (F/χ²)	P value
RH ARR Group	-	-	A	B	C	D	-	-
Number (%) of subjects	-	-	40 (35%)	22 (19%)	29 (25%)	24 (21%)	-	-
Baseline	-	-	-	-	-	-	-	-
Age	-	years	54 ± 19	61 ± 14	65 ± 14	62 ± 12	F(3, 115) = 3.12	0.03
PRA	0.10-6.56	ng/ml/hr	5.20 ± 6.81	0.80 ± 0.40	0.57 ± 0.31	0.26 ± 0.14	F(3, 115) = 11.62	1E-6
S Aldosterone	2.52-39.2	ng/dL	10.73 ± 6.43	12.08 ± 6.07	14.33 ± 6.20	20.91 ± 8.61	F(3, 115) = 11.87	8E-7
ARR	-	-	4.31 ± 2.52	15.46 ± 3.08	28.06 ± 6.48	92.39 ± 51.22	F(3, 115) = 74.37	1E-26
PRA suppressed, n (%)	<0.65	ng/ml/h	2 (5%)	8 (36%)	20 (69%)	23 (96%)	χ²(3, 115) = 58.02	< 1E-5
PRA suppressed, n (%)	<1.0	ng/ml/h	4 (10%)	15 (68%)	24 (83%)	24 (100%)	χ²(3, 115) = 59.92	< 1E-5
HTN duration	-	years	12.3 ± 13.2	11.1 ± 10.0	15.4 ± 7.6	12.8 ± 8.5	F(3, 115) = 0.81	0.49
SBP highest	90-120	mmHg	179 ± 26	161 ± 37	188 ± 15	168 ± 30	F(3, 115) = 4.96	0.003
DBP highest	60-80	mmHg	97 ± 20	103 ± 20	109 ± 27	100 ± 8	F(3, 115) = 2.04	0.11
Number of HTN meds (max)		-	3.95 ± 0.77	3.75 ± 0.43	3.71 ± 0.45	3.71 ± 0.45	F(3, 115) = 1.37	0.25
Pulse rate	60-100	per min	90 ± 20	84 ± 17	76 ± 11	83 ± 9	F(3, 115) = 4.56	0.005
S Creatinine	0.55-1.18	mg/dl	0.81 ± 0.18	0.82 ± 0.24	0.85 ± 0.33	0.88 ± 0.31	F(3, 115) = 0.41	0.75
eGFR	-	ml/minute/1.73m^2^	92.9 ± 21.4	91.4 ± 18.4	88.2 ± 14.7	83.4 ± 18.0	F(3, 115) = 1.43	0.16
Urine A:C ratio	<30	µg/mg	144 ± 266	131 ± 200	353 ± 759	234 ± 253	F(3, 115) = 1.60	0.19
S Sodium	135-150	mEq/L	137.7 ± 3.0	138.0 ± 3.9	136.1 ± 4.4	138.4 ± 4.3	F(3, 115) = 1.88	0.14
S Potassium	3.5-5.4	mEq/L	4.67 ± 0.46	4.75 ± 0.57	4.75 ± 0.45	4.48 ± 0.48	F(3, 115) = 1.69	0.17
Adrenal CT normal (%)	-	-	-	-	29 (100%)	13 (56%)	χ²(1, 53) = 13.46	2E-4
Adrenal CT abnormal (%)	-	-	-	-	0 (0%)	11 (44%)	χ²(1, 53) = 13.46	2E-4
Current/Follow-Up	-	-	-	-	-	-	-	-
Number of HTN meds (current)	-	-	2.90 ± 0.81	2.78 ± 0.42	3.00 ± 0.67	2.67 ± 0.85	F(3, 115) = 1.03	0.38
MRA therapy, n (%)	-	-	1 (3%)	6 (27%)	12 (41%)	12 (50%)	χ²(3, 115) = 21.68	< 1E-4
MRA withdrawn, n (%)	-	-	0 (0%)	4 (17%)	2 (8%)	8 (33%)	χ²(3, 115) = 14.10	0.003
SBP current	90-120	mmHg	144 ± 22	139 ± 16	126 ± 29	130 ± 21	F(3, 115) = 4.15	0.008
DBP current	60-80	mmHg	87 ± 17	82 ± 13	77 ± 11	84 ± 9	F(3, 115) = 3.19	0.026
Comorbidities	-	-	-	-	-	-	-	-
ECHO EF	50-70	%	60.5 ± 4.2	61.2 ± 3.4	59.7 ± 5.3	59.3 ± 2.7	F(3, 115) = 1.03	0.15
IHD, n(%)	-	-	4 (10%)	0 (0%)	3 (10%)	4 (17%)	-	-
Stroke (%)	-	-	2 (5%)	0 (0%)	4 (14%)	0 (0%)	-	-
Atrial fibrillation (%)	-	-	2 (5%)	0 (0%)	1 (3%)	0 (0%)	-	-
OSA (%)	-	-	4 (20%)	2 (20%)	4 (27%)	1 (7%)	-	-
AHI	< 5	-	21.7 ± 21.6	12.3 ± 6.5	13.7 ± 9.5	15.0 ± 3.0	F(3, 60) = 1.52	0.22

ARR screening

**Figure 1:**
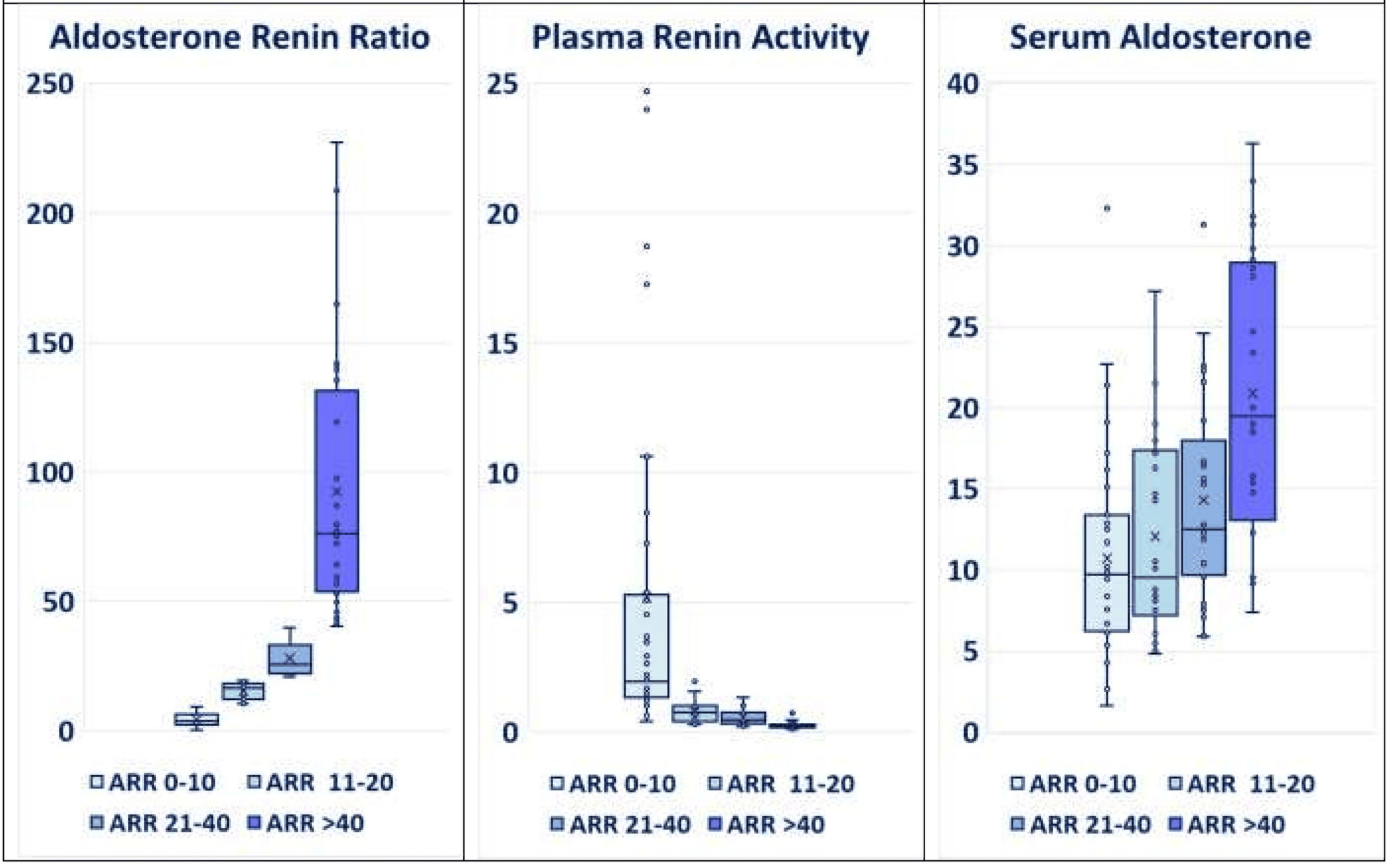
Aldosterone renin ratio (ARR), plasma renin activity (PRA), and serum aldosterone in patients with resistant hypertension evaluated in the study, grouped according to increasing ARR (N= 115). Box and Whisker plots of the four ARR groups. ANOVA P values: PRA= 1E-6; SA= 8E-7; ARR= 1E-26. (Please note the absence of any sharp diagnostic cut-offs between ARR 11 – 20, 21 – 40, and > 40). The ARR values among the patients varied widely, ranging from 0.4 to 227. The distribution of ARR values is detailed herewith, categorizing the patients into distinct groups to facilitate analysis (Group A: ARR < 10: 35% of patients; Group B: ARR 11-20: 19%; Group C: ARR 21-40: 25%; and Group D: ARR > 40: 21%). Overall, 46% of the patients had an ARR >20, indicating a positive screen for primary aldosteronism. ARR: aldosterone renin ratio; PRA: plasma renin activity

**Figure 2:**
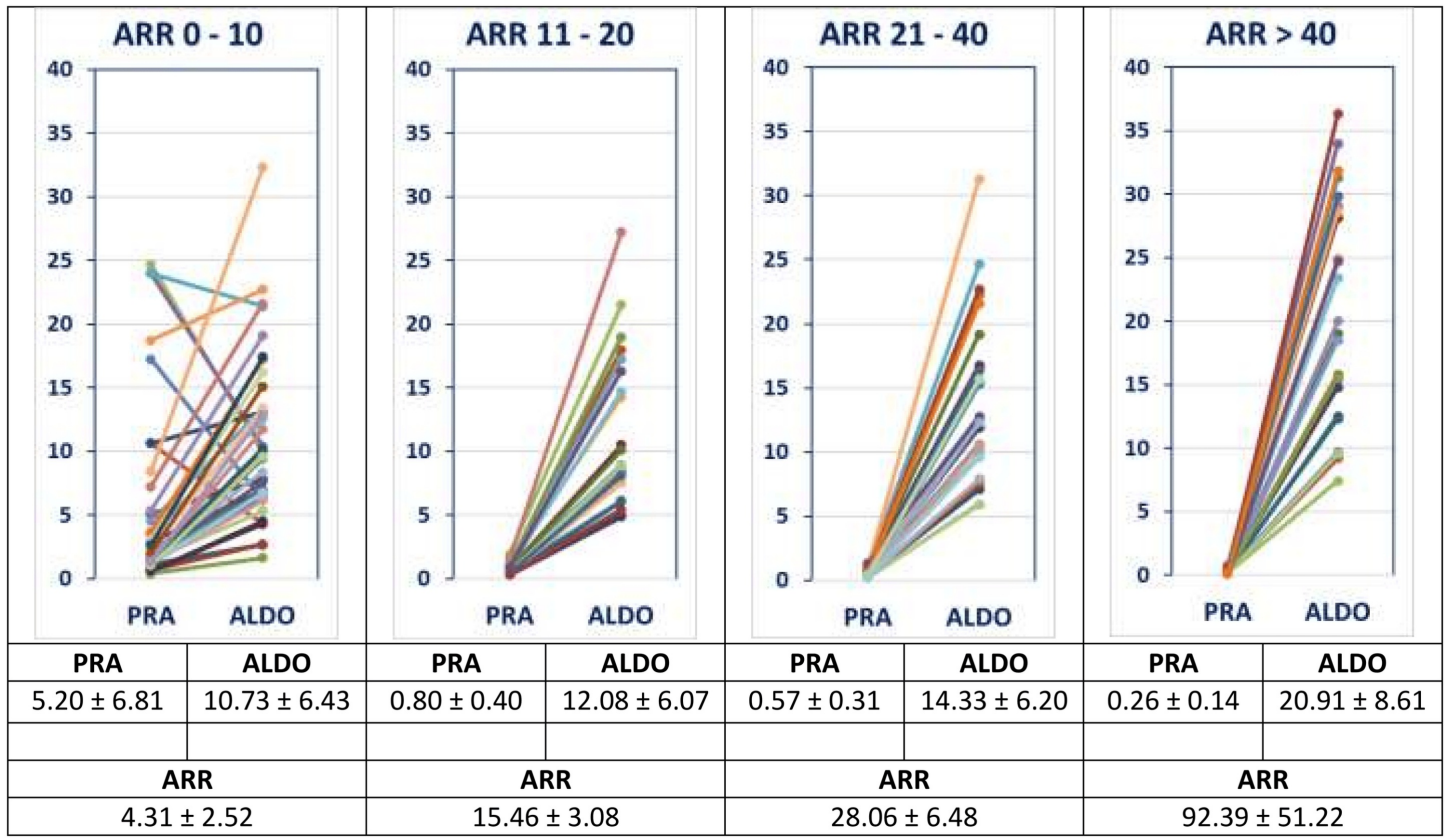
The spectrum/continuum of pre-primary and primary aldosteronism: relationship between PRA and serum aldosterone in patients evaluated for resistant hypertension, grouped according to increasing ARR (N= 115) ANOVA P values: PRA= 1E-6; SA= 8E-7; ARR= 1E-26. (Please note the absence of any sharp diagnostic cut-offs between ARR 11 – 20, 21 – 40 and > 40). PRA: plasma renin activity; ARR: aldosterone renin ratio; ALDO: serum aldosterone

The distribution of ARR values is detailed in Table 1 and Figures 1, 2, which categorize the patients into distinct groups to facilitate analysis (Group A: ARR < 10; Group B: ARR 11-20; Group C: ARR 21-40; and Group D: ARR > 40). Overall, 46% of the patients had an ARR >20, indicating a positive screen for PA.

ARR comparative group analysis

**Figure 3:**
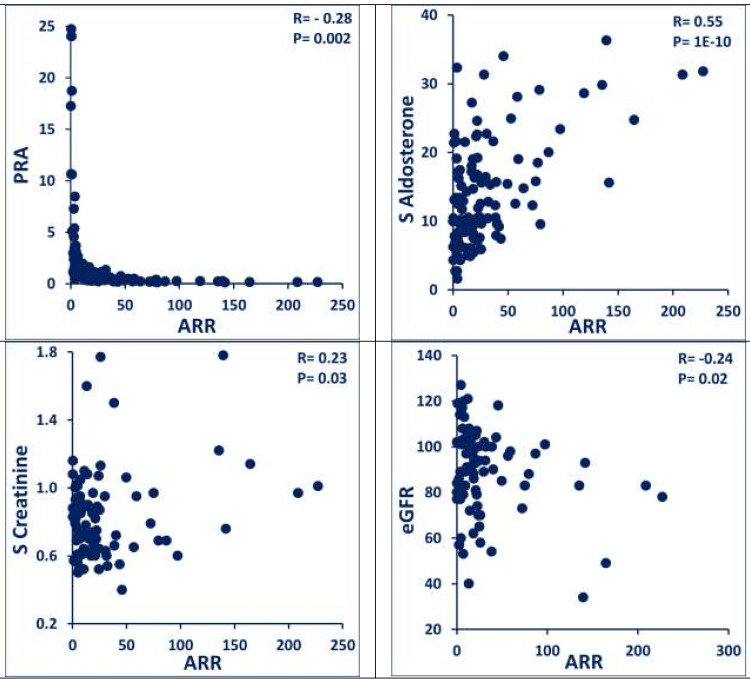
Correlations of selected endocrine metabolic biomarkers in patients with resistant hypertension evaluated in the study (N= 115) Increasing ARR tended to be associated with increase in serum creatinine (R= 0.23; P= 0.03) and decrease in eGFR (R= -0.24; P= 0.02) and increase in urine albumin:creatinine ratio. ARR: aldosterone renin ratio; S: serum; eGFR: estimated glomerular filtration rate; PRA: plasma renin activity

ARR values ranged from 0.4 to 227 (ARR <10 (35%); 11-20 (19%); 21-40 (25%); and >40 (21%)), with corresponding stepwise decreasing PRA (P= 1E-6) and increasing serum aldosterone (P= 8E-7) (Table 1; Figures 1, 2). Increasing ARR tended to be associated with an increase in serum creatinine (R= 0.23; P= 0.03), decrease in eGFR (R= -0.24; P= 0.02) and an increase in urine albumin:creatinine ratio (Table 2; Figure 3). The ARR> 40 group displayed the highest serum creatinine, lowest eGFR, higher urine albumin:creatinine ratio, highest serum sodium, lowest serum potassium, and highest (44%) abnormal adrenal imaging (bilateral hyperplasia diffuse/nodular; solitary adenoma), reflecting a later stage of the pathological spectrum (Table 1). PA treatment with MRAs had a salutary effect.

**Table 2:** Clinical and biomarker correlations in patients with resistant hypertension evaluated in the study (N= 115) Reference ranges: PRA (ng/ml/hour): lying position: 0.15-2.33, standing position: 0.10-6.56; S Aldosterone (ng/dL): upright: 2.52-39.2, supine: 1.76-23.2; S Creatinine (mg/dl): women 0.55-1.02, men 0.72-1.18 R value= Correlation coefficient. P value= P<0.05 was considered statistically significant; P≥0.05 not significant. PRA: plasma renin activity; S: serum; eGFR: estimated glomerular filtration rate; HTN: hypertension; SBP: systolic blood pressure; DBP: diastolic blood pressure; A:C: albumin:creatinine; ECHO EF%: echocardiography ejection fraction %; ARR: aldosterone renin ratio

Biomarker	Reference Range	Units	ARR versus		PRA versus		Serum Aldosterone versus	
All (N= 115)	-	-	R value	P value	R value	P value	R value	P value
Baseline	-	-	-	-	-	-	-	-
Age	-	years	0.14	0.13	-0.35	0.0001	-0.14	0.15
PRA	0.10-6.56	ng/ml/hr	-0.28	0.002	-	-	0.01	0.90
S Aldosterone	2.52-39.2	ng/dL	0.55	1E-10	0.01	0.90	-	-
HTN duration	-	years	0.10	0.32	-0.21	0.05	-0.01	0.89
SBP highest	90-120	mmHg	-0.08	0.51	0.10	0.45	-0.19	0.13
DBP highest	60-80	mmHg	0.04	0.75	0.01	0.91	0.11	0.38
S Creatinine	0.55-1.18	mg/dl	0.23	0.03	0.05	0.66	0.13	0.24
eGFR	-	ml/minute/1.73m^2^	-0.24	0.02	0.05	0.63	-0.04	0.69
Urine A:C ratio	<30	µg/mg	0.04	0.74	-0.13	0.27	0.02	0.86
S Sodium	135-150	mEq/L	-0.05	0.66	0.10	0.36	0.05	0.64
S Potassium	3.5-5.4	mEq/L	-0.06	0.59	-0.13	0.26	-0.16	0.15
Current/Follow-Up	-	-	-	-	-	-	-	-
SBP current	90-120	mmHg	-0.12	0.36	-0.04	0.77	0.10	0.46
DBP current	60-80	mmHg	-0.09	0.52	0.3	0.02	0.17	0.23
Comorbidities	-	-	-	-	-	-	-	-
ECHO EF%	50-70	%	-0.14	0.22	0.15	0.20	0.01	0.93

Adrenal imaging (CT adrenal protocol)

Among RH patients with ARR 21-40 (Group C), adrenal imaging was reported as normal in 100%. On the other hand, among RH patients with ARR > 40 (Group D), adrenal imaging was reported as normal in 56% and abnormal in the remaining 44% (spectrum of abnormalities included bilateral hyperplasia nodular: 74%, bilateral hyperplasia diffuse: 13%; solitary adenoma: 13%) (Table 1).

Additional investigations

For economic, practical, clinical, and logistical (lack of local/regional expertise in adrenal venous sampling) considerations, none of the subjects with RH and ARR > 20 were subjected to any further confirmatory endocrine testing for PA.

Therapy of PA in RH

In the absence of contraindications (hyperkalemia and/or renal dysfunction), selected patients with RH were initiated on increasing doses of MRA (spironolactone 25-200 mg/day). MRA had to be withdrawn in a subset of these subjects due to the subsequent development of significant hyperkalemia and/or renal dysfunction. Improved blood pressure control, with a parallel reduction in the number of hypertension medications (and/or their dosages), was observed in the MRA-treated cohort.

On the latest follow-up, the ARR 0-10 group of patients with RH (likely harboring pathologies, other than PA) exhibited the highest current systolic and diastolic blood pressures (Table 1), compared to the higher ARR groups with evidence of autonomous increased "renin-independent aldosterone production".

Comorbidities and cardiovascular outcomes

Comorbidities, including ischemic heart disease (IHD), cerebrovascular disease, atrial fibrillation, and obstructive sleep apnea, were examined (medical record review) across the ARR groups (Table 1).

Additional clinical presentations of PA

During the study period, in addition to presentation with RH, hypokalemia was a notable presenting symptom in four patients. In two patients, acute quadriparesis (periodic paralysis) was the presenting feature; one was subsequently identified to have adrenal carcinoma (with fatality within a year of diagnosis); the other was identified to have familial PA. PA was diagnosed in one patient who displayed accelerated hypertension and hypertensive urgency with hypokalemia (“normal” adrenals reported on CT); the surprising development of severe hyponatremia on low dose (50 mg/day) of spironolactone led to documentation of coincidental severe adrenocorticotropic hormone (ACTH) deficiency and panhypopituitarism, with empty sella. PA was suspected in the intensive care unit, and subsequently confirmed (solitary adrenal adenoma), in one critically ill patient admitted with severe bacterial sepsis, septic shock, hyperlactatemia, hypokalemia, and paradoxical alkalosis. In two normokalemic patients, PA was diagnosed during guideline-driven routine work-up for adrenal incidentalomas.

## Discussion

Our clinic-based study involving 115 patients with RH, revealed a substantial prevalence of elevated ARR values (>20) in 46% of the cohort. This is much higher than the reported prevalence of PA in the literature (i.e. epidemiological studies estimating PA prevalence at approximately 20% among patients with RH, 10% in those with severe hypertension, and 6% in individuals with otherwise uncomplicated hypertension) [1-6]. In our study, ARR values ranged from 0.4 to 227 (ARR <10 (35%); 11-20 (19%), 21-40 (25%), and >40 (21%), with corresponding stepwise decreasing plasma renin activity and increasing serum aldosterone. Increasing ARR tended to be associated with an increase in serum creatinine and decrease in eGFR and an increase in urine albumin:creatinine ratio. ARR> 40 group displayed the highest serum creatinine, lowest eGFR, higher urine albumin:creatinine ratio, highest serum sodium, lowest serum potassium, and highest (44%) abnormal adrenal imaging (bilateral hyperplasia diffuse/nodular; solitary adenoma), reflecting a later stage of the pathological spectrum. PA treatment with MRAs had a salutary effect.

"Pre-PA" or “subclinical” PA: clinical care concepts

Given the continuum nature of PA pathology and hence the arbitrariness and artificial nature of currently used ARR thresholds (positive screening= 20 to 30 ng/dL: ng/mL/hour using PRA for calculation), it may be possibly worthwhile to mark lower ARR thresholds (eg: 11-20 or 16-20; especially with suppressed renin) as pre-primary aldosteronism (akin to prediabetes and prehypertension). This clinically pragmatic and prudent approach can enhance early disease detection (renin-independent aldosterone production), monitor progression (periodic rescreening), optimize treatment, and mitigate end-organ damage in RH. This will enable flagging of patients at future risk of disease progression to “overt” PA, and incorporating early empirical MRA therapy for “essential” hypertension. Clearly, further longitudinal studies in RH cohorts from various populations are needed to validate these observations and fully elucidate the clinical course of the disease.

In our study, this PPA (ARR 11-20) group, when compared to the ARR <10 group, exhibited several significant clinical and biochemical differences (Table 1). Specifically, patients in the PPA group demonstrated: (a) significantly lower PRA levels; (b) a markedly higher prevalence of suppressed renin (defined as PRA <0.65-1.0 ng/mL/hour); (c) significantly elevated diastolic blood pressure; and (d) a slight trend toward higher serum creatinine. These data support the hypothesis that patients in this group are at an earlier stage of the disease spectrum, facilitating (through serial monitoring) early identification, leading to prompt interventions and potential reduction of long-term cardiovascular and renal complications of PA. Perhaps, we should avoid using the term “subclinical” for this stage, since there is already evidence of early organ dysfunction/damage at this early stage itself. These observations further reinforce the recent concept of PA as a spectrum disease (renin-independent aldosterone production).

**Figure 4:**
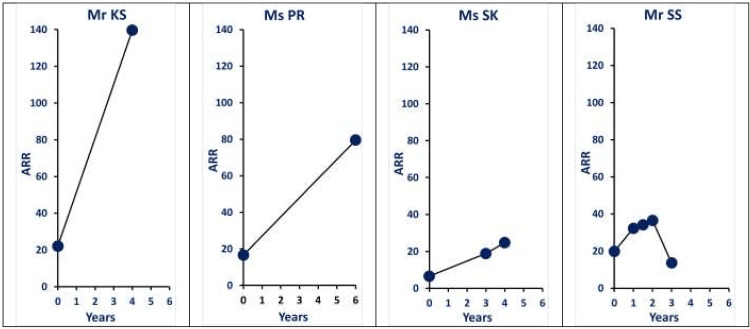
Serial ARR measurements: examples of documentation of progression of primary aldosteronism (renin-independent aldosterone production) Mr KS: A 84-year-old male patient with resistant hypertension. (Adrenal imaging: Adrenal glands are normal in size, shape and attenuation values.) Ms PR: A 71-year-old female patient with resistant hypertension. (Adrenal imaging: Small hypodense lesion 5.6 x 6.2 mm, within the medial limb of the left adrenal gland.) Ms SK: A 64-year-old female patient with resistant hypertension (Adrenal imaging: CT adrenals ordered; Ultrasound adrenal areas normal). Mr SS: A 68-year-old male patient, with no history of hypertension (Adrenal imaging: 20 x 19 mm incidentaloma; HU -12). Recognizing the continuum nature of PA and marking the newly proposed stage of “Pre-Primary Aldosteronism” (ARR 11-20) can enhance early disease detection (renin-independent aldosterone production), monitor progression (periodic rescreening), optimize treatment, and mitigate end-organ damage in RH. ARR: aldosterone renin ratio; RH: resistant hypertension; PA: primary aldosteronism

A few examples of clinical documentation of the progression of PA (renin-independent aldosterone production) are illustrated in Figure 4.

Severe PA

Similarly, patients with an ARR >40, who can be categorized as having severe PA, exhibited a more advanced phenotype with notable biochemical and imaging findings. This group displayed a consistent trend toward more pronounced renal impairment, as reflected by higher serum creatinine levels, lower eGFR, and elevated urine albumin-to-creatinine ratios, indicative of early nephropathy. Additionally, these patients exhibited a tendency towards higher serum sodium levels and lower serum potassium levels, though both remained within normal physiological ranges. Most interestingly, abnormal adrenal imaging was exclusive to this group, with 44% of patients displaying adrenal pathology (vide supra). This contrasts with the ARR 21-40 group, where 100% of patients had normal adrenal imaging, suggesting that severe PA represents a more advanced stage in the PA spectrum.

Limitations of ARR screening for PA and role of confirmatory endocrine testing

Despite some of the well-known limitations (lower specificity, despite high sensitivity) of ARR screening, hailing from a resource-limited setting, we had to choose a pragmatic and practical approach to diagnosing and treating PA in patients with RH. However, the most accurate results for the ARR require strict adherence to fasting, posture control, and medication adjustments (Table 3). For economic, practicalm clinical, and logistical (lack of local/regional expertise in adrenal venous sampling) considerations, none of the subjects with RH and ARR> 20 in our study were subjected to any further confirmatory endocrine testing for PA.

The Endocrine Society recommends drawing blood for an ARR test in the morning after the patient has been out of bed for at least two hours and seated for 5-15 minutes. However, we chose a more stringent protocol (Table 3; correspondingly theoretically more discriminative) at our clinic, especially since any further confirmatory endocrine testing for PA was not feasible in the prevailing circumstances.

**Table 3:** Overview of protocols for drawing blood for ARR screening test for primary aldosteronism: Influence of patient preparation, posture, and sampling conditions Recommendations Based on Guidelines: 1. Endocrine Society Guidelines recommend a stringent approach for the most accurate assessment, particularly in high-risk patients. 2. European Society of Hypertension (ESH) allows for more flexibility depending on the clinical setting and patient needs. Practical consideration and conclusion: While the stringent protocol is ideal and the most accurate results for the ARR require strict adherence to fasting, posture control, and medication adjustments, in real-world practice, a more relaxed protocol may be used for initial screening, with follow-up testing under stricter conditions if necessary. ACE: angiotensin-converting enzyme; NSAID: nonsteroidal anti-inflammatory drug; ARB: angiotensin II receptor blocker; ARR: aldosterone renin ratio

Procedure Details	Stringent Protocols (Most Accurate)	Moderate Protocols (Pragmatic Approach)	Simple Protocols (Initial Screening)	Remarks
Fasting Requirement:	Overnight (at least 8-10 hours).	Fasting is not strictly required, but light meals low in sodium are encouraged.	No fasting is required.	Food intake can affect aldosterone levels.
Posture Requirement:	-	-	-	-
Resting Period:	The patient rests in a seated or supine position for at least 1-2 hours before blood draw.	The patient rests for 30 minutes in a seated position before blood draw.	The patient’s position can be either seated or lying down, with a brief rest period (5-10 minutes) before the blood draw.	-
Position for Blood Draw:	Blood is drawn after 30 minutes of standing or walking (to standardize upright posture).	Blood is drawn while seated or standing after resting in the same position for 30 minutes.	Blood is drawn without requiring any specific posture adjustments.	This is critical because posture affects aldosterone secretion, which is higher when standing compared to lying down.
Medication Adjustments:	-	-	-	-
ACE inhibitors, ARBs, diuretics, beta-blockers, and NSAIDs:	Should ideally be discontinued for 2-4 weeks before testing.	Some medications may still need to be adjusted, but this protocol allows for more flexibility with essential medications that cannot be stopped.	Limited or no medication adjustments are made. This protocol is often used in situations where medication discontinuation is not feasible.	These medications can affect renin and aldosterone levels.
Spironolactone, eplerenone, and amiloride:	Should be stopped at least 6 weeks before testing.	-	-	Mineralocorticoid receptor antagonists and potassium-sparing diuretics.
Sample Timing:	Morning sampling is preferred.	Allows for any time of the day sampling.	Allows for any time of the day sampling.	Aldosterone levels can exhibit diurnal variation, typically peaking in the morning and decreasing throughout the day.

As discussed, when diagnosing PA, ARR is a critical screening tool, but its interpretation is highly variable due to the lack of a clear consensus on cutoff values. The definition of PA and the ARR cutoff used to screen for it often depend on the specific assays used and the units of measurement (e.g., ng/dL for aldosterone and ng/mL/hour for renin activity, or pg/mL for direct renin concentration). This variability can complicate the diagnostic process. Different studies and guidelines recommend varying ARR cutoff values for PA diagnosis. ARR cutoffs typically range from 20 to 40 depending on the units and assays used. Some centers use a cutoff of 20 ng/dL per ng/mL/hour, while others may recommend higher cutoffs (e.g., 30 or 40) to balance between sensitivity and specificity. This lack of standardization means clinicians must interpret ARR values carefully within the context of the assay employed and the clinical picture. As the ARR cutoff increases, the specificity of the test increases, meaning fewer false positives, but the sensitivity decreases, leading to potential false negatives. A higher cutoff value reduces the risk of misdiagnosing non-PA patients but increases the likelihood of missing milder cases of PA. Conversely, lower ARR cutoffs yield higher sensitivity but may produce more false positives, leading to unnecessary confirmatory testing or interventions. In clinical practice, the balance between sensitivity and specificity is crucial.

Evolving insights on adrenal imaging in PA

CT imaging plays a pivotal role in the diagnosis of PA, aiding in the differentiation of various adrenal pathologies. The variability in reported prevalence rates across studies reflects the broad spectrum of PA severity and differences in imaging protocols. As traditionally reported, solitary aldosterone-producing adenomas (APAs) (35-40%) and bilateral adrenal hyperplasia (BAHs) (20-60%; diffuse adrenal enlargement) remain the most common findings in PA. Macronodular disease (5%), micronodular disease (3-5%), unilateral adrenal hyperplasia (1-2%), and adrenal carcinoma (<1%) are other adrenal pathologies reported. Given the recognized continuum in PA pathology, ranging from mild to severe forms, the prevalence of adrenal imaging abnormalities varies depending on the diagnostic criteria used. In patients with milder PA, adrenal imaging may reveal normal or subtly abnormal findings.

Studies show that a substantial number of PA patients (20-40%) have normal adrenal CT scans, despite biochemical confirmation of excess aldosterone production. Thus, the relative distribution of various adrenal pathologies in PA may vary significantly depending on the nature of the cohort being evaluated (e.g., age groups; severity of hypertension; normokalemic versus hypokalemic patients; and other clinical characteristics) [11-13]. A recent study has rightly concluded that morphologically normal-appearing adrenal glands are commonly the source of aldosterone production in PA, even among young patients [14]. With respect to the role of functional testing and adrenal venous sampling, which is considered to be the gold standard for distinguishing unilateral from bilateral PA, the debate continues. A more nuanced approach that integrates imaging with functional assessments will emerge in the coming years.

Pathophysiology and high prevalence of PA (renin-independent aldosterone production)

The high prevalence of PA has been attributed to the diverse and multifocal nature of aldosterone production in the adrenal glands, facilitated by advanced diagnostic techniques revealing the complex pathophysiology of this condition. PA (autonomous aldosterone production) has been traditionally broadly classified into unilateral aldosterone production (APA and BHA). APAs are well-defined, tangible masses, whereas BHA represents a more complex and elusive pathology. Recent advancements in aldosterone synthase-specific antibodies and genomic sequencing have significantly enhanced our understanding of PA. Traditional diagnoses relied on basic histology of resected adrenal glands, imaging, and adrenal vein sampling. However, immunohistochemical staining has revealed a spectrum of PA pathology, including bilateral adenomas, unilateral hyperplasia, micronodules, and aldosterone-producing cell clusters (APCCs) in various combinations. These findings indicate that even within a single adrenal gland, multiple sites of aldosterone production can coexist, challenging the simplicity of APA versus BHA nomenclature.

Studies have shown that APCCs are subcapsular clusters expressing aldosterone synthase, distinct from the continuous zona glomerulosa (ZG) seen in young human and rodent adrenals. APCCs have been observed adjacent to APAs and even in normal adrenal tissues, suggesting their role in autonomous aldosterone production. An age-dependent increase in the number of APCCs has been documented, contrasting with a decline in the overall aldosterone synthase-expressing area, supporting the hypothesis that these clusters contribute to autonomous aldosterone production and the prevalence of PA [15-16].

Mild PA phenotype

Several additional lines of evidence exist towards the description of the mild PA phenotype (spectrum of MRA-responsive disease rather than a binary condition), especially in patients with RH and mildly elevated or normal aldosterone levels. This PA spectrum encompasses a range of phenotypes, from milder forms with subtle hypertension or even normal blood pressure and potassium levels to severe PA with overt hypertension and hypokalemia [17]. Several lines of evidence support this broader perspective.

Patients with PA often exhibit varying degrees of blood pressure and potassium normalization in response to MRAs [18]. Moreover, some individuals with normal blood pressure and potassium levels may still exhibit a response to MRAs, indicating the presence of subclinical PA [19]. ARR, a commonly used diagnostic test for PA, may not always be accurate in diagnosing mild or subclinical PA, especially in patients with normal blood pressure and potassium levels [20].

The Prevention and Treatment of Hypertension With Algorithm-based therapy-2 (PATHWAY-2) trial demonstrated that patients with RH responded well to MRAs, even those with ostensibly normal aldosterone levels, suggesting mechanisms beyond classic PA, such as relative aldosterone excess, MR hypersensitivity, MR activation via occult ligands, or other mechanisms [21].

Also, genetic heterogeneity plays a significant role in the spectrum of PA. Various genetic mutations can contribute to different phenotypes, and genetic factors may interact with environmental factors to influence the severity of the condition [22].

Thus, the common, often unrecognized mild phenotype of PA, has been included in the broader terminology of “renin-independent aldosterone production”, which is more often diagnosed when the PA phenotype is more severe. Thus, renin-independent aldosterone production can be detected in both normotensive and hypertensive individuals, with its severity correlating with blood pressure elevation, incident hypertension risk, cardiovascular disease, and the effectiveness of MRA therapy. It has been rightly suggested that expanding PA screening indications and employing a pathophysiology-based approach emphasizing inappropriate aldosterone production relative to renin levels will enhance diagnostic and therapeutic outcomes for PA.

Genetics of PA

PA encompasses a variety of genetic mutations that affect ion channels and pumps within adrenal cells, leading to autonomous aldosterone production. Key somatic mutations include those in the *KCNJ5* gene, which encodes the potassium channel GIRK4, and mutations in the *CACNA1D* gene, affecting calcium channels. These genetic variations lead to increased aldosterone synthase expression, contributing to hyperaldosteronism. Additionally, mutations in *ATP1A1* and *ATP2B3* genes, encoding subunits of ion pumps, have been implicated. These insights highlight the heterogeneity and complexity of PA at a molecular level, might explain why PA is so common, support the existence of milder and evolving forms of PA, and also influence both diagnosis and treatment [23].

Limitations of the study

A major limitation of our study is its retrospective, observational design, which may introduce biases in patient selection and data interpretation. As a clinic-based study, the results may not be generalizable to the broader population. Additionally, due to resource limitations, we were unable to conduct guideline-recommended confirmatory tests such as saline suppression tests or adrenal venous sampling, which could have provided more definitive diagnoses. Furthermore, the study was limited to patients with RH, leaving unexplored the prevalence and characteristics of PA in individuals with less severe forms of hypertension, where early detection might have a substantial impact.

Strengths of the study

Our study contributes to the growing body of evidence supporting the spectrum-based understanding of PA, rather than its traditional binary characterization. The study’s strength lies in its clinic-based focus on RH in a resource-limited setting, addressing the underdiagnosis of PA in such environments. Our study identified a significant portion of patients with milder elevations in ARR (with the possibility of future progression to overt PA), who may otherwise have been missed, highlighting the potential importance of screening to detect early-stage disease, allowing for timely intervention with MRAs and thereby reducing long-term end-organ damage.

Need for future studies

Prospective, long-term interventional studies are needed to validate the spectrum-based approach to PA, incorporating confirmatory testing such as adrenal venous sampling to ensure accurate diagnoses. In resource-limited settings, the development of simplified and cost-effective screening and confirmatory testing protocols could greatly improve detection and treatment. Moreover, exploring the role of genetic factors in the variability of aldosterone production and the progression from “pre-primary” to overt PA could provide deeper insights into disease pathogenesis.

## Conclusions

The significance of PA in contributing to RH is highlighted and the importance of early detection in resource-limited settings is emphasized. Our observations further reinforce that PA is not a binary condition, but exists as a spectrum disorder responsive to MRAs, even in patients with mildly elevated or normal aldosterone levels. Early disease detection/ recognition (renin-independent aldosterone production) can be facilitated by marking “pre-PA” (ARR 11-20), followed by monitoring progression (periodic rescreening) and optimizing treatment. Analysis of ARR values demonstrated an association between higher ARR and adverse renal markers, such as increased serum creatinine and decreased eGFR along with elevated urine albumin ratios. Additionally, adrenal imaging abnormalities were more prevalent in patients with ARR >40, indicating advanced PA stages.

Thus, renin-independent aldosterone production can be detected in individuals with normotension, mild hypertension, as well as, RH, with corresponding superior effectiveness of MRA therapy. Expanding PA screening indications and employing a pathophysiology-based approach emphasizing inappropriate aldosterone production relative to renin levels will enhance diagnostic and therapeutic outcomes for PA. A practical, pragmatic and prudent approach is essential, especially in resource limited milieu, to address the vastly underdiagnosed and undertreated medical challenge of PA, with hopeful mitigation of end-organ damage.

## References

[1] Jaffe G, Gray Z, Krishnan G (2020). Screening rates for primary aldosteronism in resistant hypertension: a cohort study. Hypertension.

[2] Douma S, Petidis K, Doumas M (2008). Prevalence of primary hyperaldosteronism in RH: a retrospective observational study. Lancet.

[3] Nishikawa T, Omura M, Satoh F, Shibata H, Takahashi K, Tamura N, Tanabe A (2011). Guidelines for the diagnosis and treatment of primary aldosteronism--the Japan Endocrine Society 2009. Endocr J.

[4] Byrd JB, Turcu AF, Auchus RJ (2018). Primary aldosteronism: practical approach to diagnosis and management. Circulation.

[5] Bioletto F, Bollati M, Lopez C (2022). Primary aldosteronism and resistant hypertension: a pathophysiological insight. Int J Mol Sci.

[6] Funder JW, Carey RM, Mantero F (2016). The management of primary aldosteronism: case detection, diagnosis, and treatment: an Endocrine Society clinical practice guideline. J Clin Endocrinol Metab.

[7] Vaidya A, Carey RM (2020). Evolution of the primary aldosteronism syndrome: updating the approach. J Clin Endocrinol Metab.

[8] Memon SS, Bandgar T (2024). Update on primary aldosteronism: time to screen all hypertensives. J Assoc Physicians India.

[9] Memon SS, Lila A, Barnabas R (2022). Prevalence of primary aldosteronism in type 2 diabetes mellitus and hypertension: a prospective study from Western India. Clin Endocrinol (Oxf).

[10] Alam S, Kandasamy D, Goyal A, Vishnubhatla S, Singh S, Karthikeyan G, Khadgawat R (2021). High prevalence and a long delay in the diagnosis of primary aldosteronism among patients with young-onset hypertension. Clin Endocrinol (Oxf).

[11] Young WF, Stanson AW, Thompson GB, Grant CS, Farley DR, van Heerden JA (2004). Role for adrenal venous sampling in primary aldosteronism. Surgery.

[12] Rossi GP, Bernini G, Caliumi C (2006). A prospective study of the prevalence of primary aldosteronism in 1,125 hypertensive patients. J Am Coll Cardiol.

[13] Rossi GP (2011). A comprehensive review of the clinical aspects of primary aldosteronism. Nat Rev Endocrinol.

[14] Parksook WW, Yozamp N, Hundemer GL (2022). Morphologically normal-appearing adrenal glands as a prevalent source of aldosterone production in primary aldosteronism. Am J Hypertens.

[15] Williams TA, Reincke M (2022). Pathophysiology and histopathology of primary aldosteronism. Trends Endocrinol Metab.

[16] Lim JS, Rainey WE (2020). The potential role of aldosterone-producing cell clusters in adrenal disease. Horm Metab Res.

[17] Vaidya A, Mulatero P, Baudrand R, Adler GK (2018). The expanding spectrum of primary aldosteronism: implications for diagnosis, pathogenesis, and treatment. Endocr Rev.

[18] Stavropoulos K, Papadopoulos C, Koutsampasopoulos K, Lales G, Mitas C, Doumas M (2018). Mineralocorticoid receptor antagonists in primary aldosteronism. Curr Pharm Des.

[19] Brown JM, Robinson-Cohen C, Luque-Fernandez MA (2017). The spectrum of subclinical primary aldosteronism and incident hypertension: a cohort study. Ann Intern Med.

[20] Bioletto F, Lopez C, Bollati M (2023). Predictive performance of aldosterone-to-renin ratio in the diagnosis of primary aldosteronism in patients with resistant hypertension. Front Endocrinol (Lausanne).

[21] Williams B, MacDonald TM, Morant S (2015). Spironolactone versus placebo, bisoprolol, and doxazosin to determine the optimal treatment for drug-resistant hypertension (PATHWAY-2): a randomised, double-blind, crossover trial. Lancet.

[22] Scholl UI (2022). Genetics of primary aldosteronism. Hypertension.

[23] Zennaro MC, Boulkroun S, Fernandes-Rosa FL (2020). Pathogenesis and treatment of primary aldosteronism. Nat Rev Endocrinol.

